# Evaluation of Parameters Relevant to Paraclinoid Microsurgery: Morphometric Analysis of the Anterior, Middle, and Posterior Clinoid Processes

**DOI:** 10.3390/tomography12100140

**Published:** 2026-09-24

**Authors:** Elif Sena Yaz, Nurullah Yücel, Alpay Alkan

**Affiliations:** 1 Department of Anatomy, Faculty of Medicine, Istanbul Atlas University, Istanbul 34408, Türkiye; 2 Department of Anatomy, Hamidiye Faculty of Medicine, University of Health Sciences, Istanbul 34668, Türkiye; nurullah.yucel@sbu.edu.tr; 3 Department of Radiology, Bezmialem Foundation University Hospital, Istanbul 34093, Türkiye; aalkan@bezmialem.edu.tr

**Keywords:** computed tomography angiography, morphometry, paraclinoid region, anterior clinoid process, skull base

## Abstract

**Background/Objectives:** The paraclinoid region—bounded by the anterior clinoid process (ACP), middle clinoid process (MCP), posterior clinoid process (PCP), optic strut (OS), and sella turcica—is critical in skull base anatomy owing to its proximity to the optic nerve, internal carotid artery (ICA), and cavernous sinus. Because the bony anatomy of this region varies considerably between individuals, detailed morphometric characterization is a prerequisite for anticipating the working corridor before anterior clinoidectomy and related approaches. This study aimed (1) to quantify the morphometric parameters of the optic canal (CO) and the ACP, MCP, and PCP using computed tomography angiography (CTA), (2) to test right–left and sex-based differences and topographic correlations among these parameters and cranial dimensions, and (3) to derive exploratory morphometric classifications of ACP shape and the ACP–OS relationship. **Methods:** High-resolution CTA images from 102 patients (204 sides) were retrospectively analyzed. Bilateral morphometric measurements were obtained for the CO and the ACP, MCP, and PCP, together with bilateral and ipsilateral interclinoid distances and cranial (biparietal, fronto-occipital) diameters. Data were analyzed with descriptive statistics, right–left and sex-stratified comparisons, and Pearson correlations. ACP pneumatization was classified into four types, and three exploratory, data-derived classifications—the Anteroclinoid Morphometric Index (AMI), ACP Size Score (ASS), and Paraclinoid Access Index (PAI)—were constructed from the linear measurements. **Results:** ACP basal width, length, ACP–OS distance, and CO-level basal width were symmetric and sex-independent, whereas the ACP–PCP and ACP–MCP distances were significantly greater on the left (*p* = 0.015 and *p* < 0.001), a pattern more pronounced in men. ACP pneumatization was most commonly absent (Type 0; 79.4% right, 82.4% left) and did not differ by side or sex. Bilateral interclinoid distances correlated with one another and, more strongly in men, with biparietal diameter. The ASS and, more weakly, the PAI correlated with the ipsilateral ACP–MCP distance (right ASS–ACP-MCP r = 0.382, *p* < 0.001; left r = 0.312, *p* = 0.001) and with CO transverse diameter, chiefly on the left (ASS r = 0.232, *p* = 0.019; PAI r = 0.352, *p* < 0.001), indicating that these indices track broader topographic variation in the paraclinoid complex rather than the ACP alone. The predominant bilateral categories were AMI-2 (39.2%), ASS-2 (31.4%), and PAI-2 (61.8%). **Conclusions:** ACP, MCP, and PCP morphometry in this cohort was largely symmetric, with a consistent left-sided predominance restricted to the ACP–PCP and ACP–MCP distances. The AMI, ASS, and PAI are exploratory, data-derived classifications—not validated surgical indices—that provide a quantitative framework for describing ACP morphology and its relationship to the optic strut; whether they carry independent value for predicting surgical corridor width or complication risk has not been tested against operative outcomes and requires prospective validation.

## Simple Summary

The paraclinoid region is a complex area at the base of the skull that contains important structures such as the optic nerve and internal carotid artery. Small differences in the shape and position of the bony structures in this region may be relevant to skull base surgery. In this study, computed tomography angiography images from 102 individuals were examined to measure the anterior, middle, and posterior clinoid processes and their relationships with the optic strut. Most measurements of the anterior clinoid process were similar on the right and left sides, whereas some distances between the clinoid processes were greater on the left side. Pneumatization of the anterior clinoid process was uncommon and was most frequently absent. We also developed three exploratory morphometric classifications—the Anteroclinoid Morphometric Index, ACP Size Score, and Paraclinoid Access Index—to describe the size and shape of these structures and their possible relationship to the surgical corridor. These findings provide anatomical reference data that may be useful for preoperative assessment in the paraclinoid region, pending further clinical validation.

## 1. Introduction

A thorough understanding of skull base anatomy is essential given the complex, closely packed arrangement of neurovascular and osseous structures in this region. The paraclinoid region in particular—the segment of the anterior and middle cranial fossa bounded by the anterior clinoid process (ACP), middle clinoid process (MCP), posterior clinoid process (PCP), optic strut (OS), optic canal, and sella turcica—is one of the most anatomically demanding areas in cranial base surgery. The pituitary gland, internal carotid artery (ICA), optic nerve, and cavernous sinus lie in extremely close proximity to one another within and around this space, so that surgical corridors through it must be planned around millimeter-scale bony landmarks [1,2].

Cross-sectional anatomy of the paraclinoid complex. The ACP is a pyramid-shaped projection of the lesser sphenoid wing that forms part of the roof of the optic canal, the anterior extent of the cavernous sinus roof, and a key attachment point of the anterior petroclinoid (Gruber’s) and interclinoid dural folds [1,3]. Its inferomedial surface is directly related to the clinoidal and ophthalmic segments of the ICA as the artery transitions from the cavernous sinus to the subarachnoid space at the anterior genu—the anatomical basis for classifying paraclinoid (“carotid–ophthalmic”) aneurysms by their relationship to the ACP and the two dural rings [2]. The MCP, when present, arises from the floor of the sella turcica lateral to the pituitary fossa and, together with the ACP, may bridge to form a caroticoclinoid foramen that partially encases the clinoidal ICA; the PCP projects from the dorsum sellae posterolateral to the sella. The optic strut connects the ACP to the body of the sphenoid, separating the optic canal superomedially from the superior orbital fissure and carotid groove inferolaterally, and is a key structure removed during extradural anterior clinoidectomy to mobilize the ACP and unroof the optic canal [3]. Medial to this complex, the sella turcica houses the pituitary gland, and lateral to it lies the cavernous sinus, whose superomedial wall is formed in part by the base of the ACP [4]. Because anterior clinoidectomy is performed by progressively drilling or removing these bony projections to decompress the optic nerve and expose the clinoidal ICA, the size, shape, and pneumatization of the ACP—together with its spatial relationship to the MCP, PCP, and optic strut—directly determine the width of the available surgical corridor and the risk of injury to the adjacent neurovascular structures [1,2].

In recent years, endoscopic and microsurgical techniques for skull base approaches to this region have advanced considerably, but their safety continues to depend on detailed preoperative anatomical knowledge and accurate morphometric measurement, since the morphology of the ACP–MCP–PCP complex and its relationship to the optic strut vary substantially between individuals and cannot be reliably inferred from generic anatomical descriptions alone [1,2]. Although numerous studies have separately reported linear measurements of the ACP [4,5,6,7,8], its pneumatization pattern [5,9,10,11], or the PCP [12,13], comparatively few have examined the ACP, MCP, and PCP together within a single analytical framework, and topographic correlations among these structures and overall cranial dimensions remain sparsely reported.

The present study was designed to address this gap. Specifically, we aimed to: (1) quantify, using high-resolution computed tomography angiography (CTA), the diameters, lengths, and inter-process distances of the ACP, MCP, and PCP at the skull base; (2) test for right–left and sex-based differences in these parameters; (3) examine topographic correlations among the interclinoid distances and between these distances and overall cranial dimensions (biparietal and fronto-occipital diameters); and (4) derive and describe exploratory, data-driven morphometric classifications of ACP shape (AMI), overall ACP size (ASS), and the ACP–optic strut relationship (PAI) that may serve as a starting point—pending outcome-based validation—for future work relating paraclinoid morphology to surgical corridor width and complication risk. A schematic overview of the relevant anatomy is provided in Figure 1 to orient the morphometric measurements described in Section 2.3.

## 2. Materials and Methods

### 2.1. Study Design and Population

This retrospective study was approved by the Scientific Research Ethics Committee of Altınbaş University (institutional approval also obtained from Bezmialem Vakıf University Hospital; protocol code 78042, approval date 10 December 2024). Brain CT angiography (CTA) images of patients scanned between 2014 and 2024 were reviewed. All examinations during this period were acquired on the same scanner using the identical acquisition protocol described in Section 2.2; no hardware, reconstruction, or protocol changes occurred over the study interval, so morphometric measurements are not confounded by scanner- or protocol-related variability. A total of 102 patients (204 sides) met the inclusion criteria and were included in the final analysis.

Inclusion criteria were age ≥18 years, absence of intracranial tumor or aneurysm, and no prior neurosurgical intervention. Patients younger than 18 years, those with an intracranial tumor or aneurysm, and those with a history of neurosurgery were excluded. Demographic data (age, sex) were retrieved from hospital records.

Sample size was estimated a priori using G*Power v3.1.9.4 (*t*-test family, difference between two independent means, two-tailed, 80% power, α = 0.05), yielding a minimum requirement of 66 subjects (33 per group) for an assumed effect size of d = 0.705. This effect size was derived from the sex-based comparison of the left-sided posterior optic canal diameter (COarkaçap-sol) reported by İlgin et al. [5] (male 0.55 ± 0.09 cm vs. female 0.49 ± 0.08 cm; Cohen’s d ≈ 0.70), the largest published morphometric CTA series of the canalis opticus and sella turcica region available at the time of protocol design and the closest available proxy for the primary paraclinoid comparisons planned in the present study. The final sample (n = 102) exceeded the estimated minimum.

### 2.2. Imaging Protocol

CTA images were acquired on a 128-slice scanner (SOMATOM go.Top, Siemens Healthineers, Erlangen, Germany) using a standardized protocol applied uniformly to all patients throughout the study period: tube voltage 120 kV, tube current 80 mAs, rotation time 0.5 s, slice thickness 1.25 mm, slice interval 0.6 mm, pitch 1.5, and a 512 × 512 matrix. Iodinated contrast medium (Iyopac 350, 350 mg I/mL; Vem İlaç San. ve Tic. A.Ş., 42 Maslak Ahi Evran Cad. No: 6, Ofis: 1, Maslak, Sarıyer, 34398 Istanbul, Türkiye) was administered for contrast-enhanced acquisitions.

### 2.3. Morphometric Parameters

For each parameter, the CT plane best demonstrating the relevant structure was identified, and all measurements were obtained bilaterally in millimeters, independently, by two observers (a radiologist and an anatomist) who were blinded to each other’s measurements at the time of acquisition. The value analyzed for each parameter and side was the arithmetic mean of the two observers’ measurements; the rationale for, and limitations of, this approach are discussed in Section 2.5 and Section 4.

On axial images, ACP basal width and length were measured; length was defined as the distance from the apex to a line drawn perpendicular to the base (Figure 2).

ACP pneumatization was assessed on coronal images and classified into four types according to the criteria of Suprasanna et al. [14], based on the extent of pneumatic air-cell extension into the ACP relative to its base and apex: Type 0, no pneumatization; Type 1, posterior (basal) pneumatization only, not extending to the apex; Type 2, anterior (apical) pneumatization, sparing the base; and Type 3, complete pneumatization of the process. Representative examples of Types 1 and 2 are shown in Figure 3.

On axial images, the bilateral ACP–ACP, MCP–MCP, and PCP–PCP distances, and the ipsilateral ACP–PCP, ACP–MCP, and MCP–PCP distances, were measured (Figure 4). The MCP was identified as a discrete bony projection from the floor of the sella turcica, lateral to the pituitary fossa, visible as a distinct osseous prominence on axial and/or coronal CT; no minimum size threshold was applied, so that rudimentary as well as fully formed MCPs were scored as present. This purely visual, threshold-free criterion—adopted because no validated size cutoff for the MCP has been established in the literature—is a likely explanation for the presence of the MCP in all 102 cases in our series, which is discussed further in Section 4.

To standardize measurements relative to overall cranial size, biparietal and fronto-occipital diameters were also measured on axial images (Figure 5).

### 2.4. Derived Indices

Three exploratory morphometric classifications were constructed from these measurements to summarize ACP shape and its relationship to the optic strut. We emphasize that these are data-derived descriptive classifications, not validated surgical indices; none has been tested against intraoperative findings, surgical difficulty, or complications, and the terminology used to label their categories (e.g., “compressed”, “standard”, “expansive”) should be understood as descriptive shorthand rather than an indication of established clinical meaning (see Section 4).

The Anteroclinoid Morphometric Index (AMI) was calculated as the basal width/length ratio and stratified into three categories based on the 25th and 75th percentiles of the pooled (right and left) distribution: AMI-1 (≤0.269), AMI-2 (0.27–0.35), and AMI-3 (≥0.35).

The ACP Size Score (ASS) was defined as the unweighted sum of ACP length, basal width, ACP–OS distance, and basal width at the CO level—four linear dimensions with no established a priori weighting scheme in the literature; they were summed with equal weight as a simple composite descriptor of overall ACP size, and this choice should be regarded as exploratory rather than empirically validated. Because right- and left-side percentile distributions were closely comparable, right-side thresholds were used as the reference for categorization on both sides: ASS-1 (≤27.569), ASS-2 (27.57–32.65), and ASS-3 (≥32.65).

The Paraclinoid Access Index (PAI) reports the ACP–OS distance itself, categorized rather than left as a continuous variable, to provide a simple ordinal descriptor of the potential anatomical corridor between the ACP and OS. We recognize that categorizing a single continuous measurement does not, by itself, add information beyond that measurement; its value, if any, lies solely in providing a simple three-tier descriptor for future studies that wish to stratify cases without reference to a continuous cutoff, and this should be weighed against the loss of information inherent to categorization. Since right- and left-side percentile distributions differed for this parameter, category thresholds were set using the midpoint of the two sides’ percentile values: PAI-1 (≤4.49 mm), PAI-2 (4.5–6.5 mm), and PAI-3 (≥6.5 mm).

We acknowledge that the three classifications were thresholded using three different statistical strategies (pooled percentiles for AMI, right-side percentiles for ASS, midpoint percentiles for PAI). This inconsistency reflects a data-driven, rather than a predefined anatomical or statistical, rationale, and is discussed as a limitation.

### 2.5. Statistical Analysis

Data were analyzed using SPSS v28.0. Descriptive statistics included mean, standard deviation, median, minimum, maximum, frequency, and percentage. Normality of distribution was assessed with the Kolmogorov–Smirnov test. Independent quantitative variables were compared using the independent-samples *t*-test or Mann–Whitney U test, and paired quantitative variables using the paired-samples *t*-test or Wilcoxon signed-rank test, as appropriate. Categorical variables were compared using the chi-square test, or Fisher’s exact test when expected cell counts were insufficient. Topographic relationships among interclinoid distances, cranial diameters, and the AMI/ASS/PAI-related measurements were assessed using Pearson correlation coefficients. A *p*-value <0.05 was considered statistically significant.

The right–left comparisons, the pneumatization and AMI/ASS/PAI concordance analyses, and the correlations between interclinoid distances and cranial diameters were prespecified in the original study protocol. The additional correlation analyses relating the ASS and PAI to the ACP–MCP distance and to CO transverse diameter were performed post hoc, in response to reviewer request, to explore topographic relationships not covered by the original analysis plan. Because AMI is a dimensionless shape ratio rather than an absolute linear distance, and because several other candidate correlations involved global cranial dimensions already shown to scale with overall skull size, we restricted formal reporting in the main text to the two distance-based indices (ASS, PAI) against the two most anatomically adjacent structures (ipsilateral ACP–MCP distance, CO transverse diameter); the complete exploratory correlation screen (126 comparisons) is provided in Supplementary Table S1. Given the number of comparisons performed across the study as a whole, findings—particularly those not prespecified—should be interpreted as hypothesis-generating; we did not apply a formal multiplicity correction (e.g., Bonferroni), consistent with recommendations that rigid correction is not always appropriate for exploratory analyses [15], but we explicitly flag the resulting increase in Type I error risk as a limitation (Section 4).

Measurement reproducibility was addressed procedurally by having two independent observers perform all measurements and using their mean, rather than statistically. No interobserver agreement statistic (e.g., intraclass correlation coefficient, ICC) was computed, because the two observers’ individual measurements were not retained separately; only their mean was recorded. This is acknowledged as a limitation in Section 4, together with the standard for reporting such statistics [16].

## 3. Results

The study included 102 subjects (57.8% male, 42.2% female) with a mean age of 59.31 ± 14.55 years (range 19–90). ACP pneumatization was most commonly absent (Type 0) bilaterally, observed in 79.4% on the right and 82.4% on the left, with Types 1–3 occurring at markedly lower and comparable frequencies on both sides (Table 1).

Descriptive statistics for ACP measurements (basal width, length, ACP–OS distance, and basal width at the CO level) are presented in Table 2, together with the ACP–MCP and MCP-PCP distances. Bilateral distances (ACP–ACP, MCP–MCP, PCP–PCP) and cranial diameters (biparietal and fronto-occipital) are reported in Table 3.

Paired *t*-tests showed no significant right-left difference in ACP basal width, length, basal width/length ratio, ACP–OS distance, or basal width at the CO level in the overall sample (all *p* > 0.05); this pattern was consistent in both sex subgroups. In contrast, the ACP–PCP and ACP–MCP distances were significantly greater on the left side than the right in the overall sample (*p* = 0.015 and *p* < 0.001, respectively), while the MCP–PCP distance did not differ significantly between sides (*p* = 0.984). On sex-stratified analysis, this left-sided predominance was significant for both distances in men (ACP–PCP *p* = 0.006; ACP–MCP *p* = 0.004), whereas in women it reached significance only for the ACP–MCP distance (*p* = 0.015) (Table 4).

Right and left ACP pneumatization types showed substantial concordance, with both sides classified as Type 0 in 71.6% of subjects. The McNemar–Bowker test showed no significant right-left asymmetry in the overall sample (χ^2^ = 2.733, df = 6, *p* = 0.841), nor in men (χ^2^ = 4.200, df = 5, *p* = 0.521) or women (χ^2^ = 2.400, df = 5, *p* = 0.791) analyzed separately, indicating that ACP pneumatization is largely bilaterally symmetric regardless of sex (Table 5).

The ACP–ACP, MCP–MCP, and PCP–PCP bilateral distances showed low-to-moderate, statistically significant positive correlations with one another (r = 0.269–0.352, *p* < 0.01), the strongest being between ACP–ACP and MCP–MCP (r = 0.352). The ACP–ACP distance correlated moderately with biparietal diameter (r = 0.409, *p* < 0.01), while MCP–MCP and PCP–PCP distances showed weaker but significant correlations with biparietal diameter (r = 0.214 and r = 0.227, *p* < 0.05). No significant correlation was found between fronto-occipital diameter and any paraclinoid bilateral measurement. On sex-stratified analysis, correlations between paraclinoid and cranial measurements were more numerous and stronger in men (ACP–MCP r = 0.514; ACP–PCP r = 0.316; MCP–PCP r = 0.340; MCP–biparietal r = 0.346; PCP–biparietal r = 0.321) than in women, in whom only the ACP–ACP/biparietal correlation reached significance (r = 0.383, *p* < 0.05) (Table 6).

The Anteroclinoid Morphometric Index (AMI), ACP Size Score (ASS), and Paraclinoid Access Index (PAI) were used to classify ACP morphology and its relationship to the OS. The predominant categories on the right side were AMI-2 (53.9%), ASS-2 (51.0%), and PAI-2 (71.6%), while the corresponding frequencies on the left side were 55.9%, 52.9%, and 79.4%, respectively (Table 7). Bilateral concordance was observed for AMI-2 in 39.2% of subjects, ASS-2 in 31.4%, and PAI-2 in 61.8%. No significant right-left difference was found for any of the three classifications on McNemar–Bowker testing (AMI: *p* = 0.660; ASS: *p* = 0.280; PAI: *p* = 0.226), indicating that these morphological and positional patterns were largely bilaterally symmetric (Table 7).

Topographic relationships (post hoc analysis). In response to the request to examine broader topographic relationships among the measured structures, we tested correlations between the ASS and PAI (as continuous, pre-categorization measurements) and two additional parameters: the ipsilateral ACP–MCP distance and the CO transverse diameter (Table 8). We were not able to test the specific relationships suggested—jugum sphenoidale width, sella turcica capacity, or optic canal stenosis—because these dimensions were not measured in the present dataset; we flag this as a direction for future work rather than substitute an unrelated analysis for the reviewer’s specific question. The ASS correlated significantly with the ipsilateral ACP–MCP distance on both sides (right r = 0.382, *p* < 0.001; left r = 0.312, *p* = 0.001), and the PAI correlated significantly with ACP–MCP distance on the right (r = 0.228, *p* = 0.021) but not the left (r = 0.103, *p* = 0.304). The CO transverse diameter correlated significantly with both the ASS (r = 0.232, *p* = 0.019) and the PAI (r = 0.352, *p* < 0.001) on the left side, with a similar but non-significant trend on the right (ASS r = 0.180, *p* = 0.070; PAI r = 0.179, *p* = 0.072) (Table 8). These findings indicate that the ASS and, to a lesser extent, the PAI are not purely local descriptors of the ACP but also track variation in adjacent paraclinoid structures (MCP position, optic canal caliber); as with the other post hoc analyses in this study, they should be regarded as hypothesis-generating.

## 4. Discussion

In this study, the morphometric relationships among the ACP, MCP, and PCP, as well as the positional relationship between the ACP and the OS, were systematically evaluated in 102 patients, and three exploratory morphometric classifications—the AMI, ASS, and PAI—were derived for descriptive purposes. ACP basal width and length, the ACP–OS distance, and the basal width at the level of the optic canal were symmetric, independent of sex or laterality, whereas the ACP–PCP and ACP–MCP distances were significantly greater on the left side. ACP pneumatization was most frequently observed as Type 0 and did not differ by side or sex.

The literature on ACP morphometry reports heterogeneous findings. Recent CT-based studies have continued to demonstrate variability in ACP morphometry, although Yıldızoğlu et al. reported no significant differences in ACP length or width according to sex or side in a pediatric population [4]. Similarly, a recent CT-based evaluation of the paraclinoid region demonstrated that ACP dimensions were associated with the degree of sphenoid sinus pneumatization [4].

The overall symmetry and sex-independence of most ACP measurements observed in our study are consistent with some but not all prior reports, suggesting that differences in sample size, age distribution, and measurement methodology may account for the discrepancies across the literature. Because no patients in this series underwent paraclinoid surgery, we cannot directly demonstrate that this symmetry improves surgical predictability or reduces complication risk; we can state only that symmetric anatomy provides a more consistent anatomical reference than asymmetric anatomy would, a hypothesis that requires testing against operative outcomes.

The predominance of Type 0 pneumatization observed in our series, independent of side and sex, is consistent with recent CT-based studies in which non-pneumatized ACPs were commonly observed, although considerable variation in pneumatization prevalence and distribution has been reported across populations [4,5,9,10,11]. These differences may be attributable to variations in sample size, demographic characteristics, imaging techniques, and pneumatization classification systems. A predominantly non-pneumatized ACP may be associated with a lower likelihood of encountering pneumatized air cells during anterior clinoidectomy; whether this translates into a measurable reduction in operative risk has not been assessed here and would require a surgical cohort.

The prevalence of the MCP varies across populations. Anukulsampan et al. reported an MCP prevalence of 42% among 400 sides examined by CT [17], compared with 100% in our series. This marked difference most likely reflects our threshold-free, purely visual definition of the MCP (Section 2.3), under which even rudimentary bony prominences were scored as present, rather than a true difference in anatomical prevalence; studies using stricter size or morphological criteria typically report substantially lower prevalence. This is a methodological limitation of the present study rather than a novel anatomical finding, and interobserver agreement for MCP identification—which we did not formally assess—would be valuable in future work using this or a more restrictive definition [16].

Morphometric data regarding the MCP remain limited despite its close anatomical relationship to the ACP. Owing to its proximity to the ACP and the paraclinoid segment of the ICA, the MCP is an important anatomical structure to consider during anterior clinoidectomy and surgical approaches to paraclinoid aneurysms and other anterior skull base lesions.

The ACP–PCP distance has generally been reported as bilaterally symmetric in the literature [6,7,12,13], whereas in our study both the ACP–PCP and ACP–MCP distances showed significant left-sided asymmetry, a finding that was particularly pronounced in men. This asymmetry, which contrasts with the literature, may be attributable to population differences or sample size and warrants further validation. Given the limited number of studies simultaneously evaluating the morphometric relationships among the ACP, MCP, and PCP, our study provides a comprehensive morphometric characterization of the paraclinoid region by incorporating all three clinoid processes into a single analytical framework and may serve as a useful anatomical reference for skull base surgery.

Bilateral distances between the clinoid processes have been reported with considerable variability across studies [7,8]; Staderini et al. [18] demonstrated that cranial-base asymmetry can be quantitatively characterized in conditions such as unilateral cranial synostosis. In our study, low-to-moderate positive correlations were found among these distances (the strongest being between ACP–ACP and MCP–MCP), along with a moderate correlation between the ACP–ACP distance and biparietal diameter; these relationships were markedly stronger in men. This finding suggests that the morphological relationship between the skull base and calvaria may be more predictable in men, and that paraclinoid measurements could serve as an ancillary parameter in forensic anthropological applications (sex determination, cranial reconstruction); however, this relationship requires validation in different populations.

Topographic relationships and the ASS/PAI as descriptors beyond the ACP. Addressing the request for a broader topographic-relationship analysis, our post hoc correlations (Table 8) showed that the ASS correlates with the ipsilateral ACP–MCP distance and, on the left, with CO transverse diameter, and that the PAI shows a weaker but partly overlapping pattern. This suggests that, although the ASS and PAI are constructed from ACP- and optic strut-specific measurements, they covary to some extent with the position of the MCP and the caliber of the optic canal, and so may partly reflect regional paraclinoid morphology rather than the ACP in isolation. We were unable to test the specific relationships proposed—with jugum sphenoidale width, sella turcica capacity, or optic canal stenosis—because these parameters were not measured in the present dataset; this remains an important direction for future prospective work that measures the paraclinoid complex alongside the jugum sphenoidale and sella turcica.

Theoretical relevance to common disorders of the paraclinoid and parasellar region. Although this study did not include patients with cavernous sinus pathology, skull base trauma, or sella turcica disorders, the morphometric relationships described here have plausible theoretical relevance to these conditions and may generate hypotheses for future clinically oriented studies. The ACP forms part of the anterosuperior wall of the cavernous sinus and lies immediately adjacent to the clinoidal ICA segment [4,19]; consequently, ACP size and its distance from the OS could influence the extent of bony removal required for extradural or endoscopic transorbital access to cavernous sinus lesions, including cavernous sinus-invasive pituitary adenomas graded by the Knosp classification [20]. In skull base trauma, fracture lines through the anterior and middle cranial fossa frequently traverse the region of the ACP, optic canal, and sella turcica, and a smaller or more pneumatized ACP–optic strut complex could theoretically be associated with a different fracture or optic nerve injury pattern, although this has not, to our knowledge, been specifically studied [21]. Similarly, in disorders that alter sella turcica capacity—including pituitary macroadenomas and other sellar or parasellar masses—the spatial relationship between the ACP/MCP complex and the sella could influence the feasibility of extended endonasal versus transcranial approaches; recent literature on clinoidal meningiomas has explicitly called for morphometric criteria to help decide when anterior clinoidectomy is warranted during resection [22]. These are, however, theoretical extrapolations from anatomical data and are explicitly flagged as such; none of them was directly tested in the present cohort.

Toward a clinical decision framework (theoretical, literature-based). In response to the suggestion that this anatomical dataset be linked more directly to surgical decision-making, we outline below a purely literature-based, hypothesis-generating framework relating the AMI/ASS/PAI categories to possible surgical corridor considerations. We emphasize that this framework has not been validated against surgical outcomes in our cohort or elsewhere, is not intended for direct clinical application, and is offered only as a starting point for future prospective studies designed with neurosurgical collaborators:

- A compact ACP configuration (AMI-1/ASS-1) combined with a narrow ACP–OS corridor (PAI-1) may correspond anatomically to a technically narrower extradural clinoidectomy window, in which case an intradural or staged approach, or additional optic canal unroofing before ACP mobilization, might theoretically reduce optic nerve traction—an approach consistent with stepwise clinoidectomy concepts described for anterior clinoidal meningiomas.
- An expansive ACP configuration (AMI-3/ASS-3) with a standard-to-expansive corridor (PAI-2/3) may correspond to more generous bony working space, potentially favoring a fully extradural clinoidectomy where indicated.
- Pneumatized ACP types (Type 1–3) may warrant additional preoperative attention to avoid inadvertent sinus entry and cerebrospinal fluid leak during drilling, regardless of the AMI/ASS/PAI category.

We reiterate that this framework is theoretical, has not been evaluated against surgical difficulty, complication rates, or any operative endpoint, and should not be used for clinical decision-making without prospective validation involving a neurosurgical team.

Recent studies have increasingly emphasized systematic morphological characterization of the ACP rather than relying solely on descriptive classifications [3]. In our study, the AMI and ASS quantitatively categorized ACP proportionality and overall size based on the basal width-to-length ratio. Our results similarly confirm a predominance of proportional/compact types (AMI-1/2, ASS-1/2) and bilateral symmetry. Likewise, the position of the OS relative to the ACP has largely been addressed in the literature through descriptive/positional classifications [4,8]; the PAI defined in our study converts this relationship into a distance-based, categorical measure. The predominant PAI-2 category in our series generally corresponds to the mid-width OS positions (Type C/D) reported as predominant in the literature. We reiterate, however, that the AMI, ASS, and PAI remain exploratory morphometric classifications: they have not been evaluated against surgical outcome, complication rate, or any other clinical endpoint, and terms describing their categories (e.g., “compressed”, “standard”, “expansive”) should not be read as implying demonstrated clinical significance.

Limitations. This study has several limitations. Its cross-sectional, single-center design, sample size, and reliance on CTA-based measurements without cadaveric or histological validation limit the generalizability of the findings. The AMI, ASS, and PAI thresholds were derived from the percentile distribution of this specific cohort using three different strategies (pooled, right-side, and midpoint percentiles, respectively) rather than from a predefined anatomical or outcome-based rationale; this is a data-driven approach whose generalizability to other populations is unknown, and the categorical labels used should not be interpreted as validated surgical categories. All morphometric measurements were obtained by two independent observers and analyzed as their mean, an approach intended to reduce single-observer measurement bias; however, because the two observers’ individual values were not retained, no formal interobserver agreement statistic (such as the intraclass correlation coefficient, reported according to standard guidelines [16]) could be calculated for the continuous measurements, nor could an agreement statistic (e.g., kappa) be calculated for the categorical assessments (pneumatization type, MCP presence). This is an important limitation for a study proposing millimeter-based thresholds, and future work should retain individual observer data to permit formal reliability analysis. The MCP was defined using a purely visual, threshold-free criterion, which likely explains its presence in 100% of our cohort—a finding markedly different from prior reports—and limits direct comparison with studies using stricter size-based definitions. The effect size (d = 0.705) used for the a priori power calculation was derived from a sex-based comparison of optic canal diameter in a related but not identical morphometric CTA series [5] rather than from a pilot comparison of the specific paraclinoid parameters (ACP, MCP, PCP measurements) targeted by the present study; this proxy-based approach to power estimation is a methodological limitation, and the achieved sample (n = 102) should be regarded as providing adequate power for the general class of morphometric comparisons performed here rather than for any single specific outcome. This study performed a large number of right-left, sex-stratified, correlational, and classification-based comparisons; only the comparisons in Table 1, Table 2, Table 3, Table 4, Table 5, Table 6 and Table 7 were prespecified, while the topographic correlations in Table 8 were exploratory and post hoc. A broader screen of AMI, ASS, and PAI against all measured distances and diameters was also performed and is reported in full in Supplementary Table S1; additional nominally significant correlations were observed but predominantly involved global cranial dimensions (e.g., biparietal, fronto-occipital diameter) rather than local paraclinoid structures, consistent with generalized craniometric scaling rather than a specific anatomical relationship. We did not apply a formal correction for multiple comparisons [15]; consequently, the risk of Type I error is increased across the study as a whole, and *p*-values—especially for the post hoc analyses—should be interpreted as hypothesis-generating rather than confirmatory. Finally, and most importantly, the clinical validity of the AMI, ASS, and PAI, and of the theoretical surgical-corridor framework proposed in this Discussion, has not been directly tested against surgical outcomes, operative difficulty, or complications in any cohort; prospective, multicenter studies that combine morphometric CTA analysis with intraoperative and outcome data—ideally involving neurosurgical co-investigators from the outset—are needed before these classifications can be considered for clinical use.

## 5. Conclusions

In this cohort of 102 patients, ACP morphometry was largely symmetric and independent of sex, whereas the ACP–PCP and ACP–MCP distances showed significant left-sided asymmetry, most pronounced in men. ACP pneumatization was most commonly Type 0 (absent) and did not differ by side or sex. Bilateral interclinoid distances correlated with one another and, more strongly in men, with biparietal diameter, and the ASS and PAI correlated with the ACP–MCP distance and CO transverse diameter, indicating that these measurements capture topographic variation extending beyond the ACP alone.

The AMI, ASS, and PAI reported here are exploratory, data-derived morphometric classifications rather than validated surgical indices. They provide a systematic, reproducible framework for describing ACP shape and its relationship to the optic strut across the ACP–MCP–PCP complex, and may serve as a starting point for future studies relating paraclinoid morphology to surgical planning. Whether they, or the theoretical corridor-selection framework outlined in the Discussion, have independent value for predicting surgical corridor width, operative difficulty, or complication risk in anterior clinoidectomy and related paraclinoid approaches remains to be established through prospective, outcome-based validation, ideally in collaboration with neurosurgical teams.

## Figures and Tables

**Figure 1:**
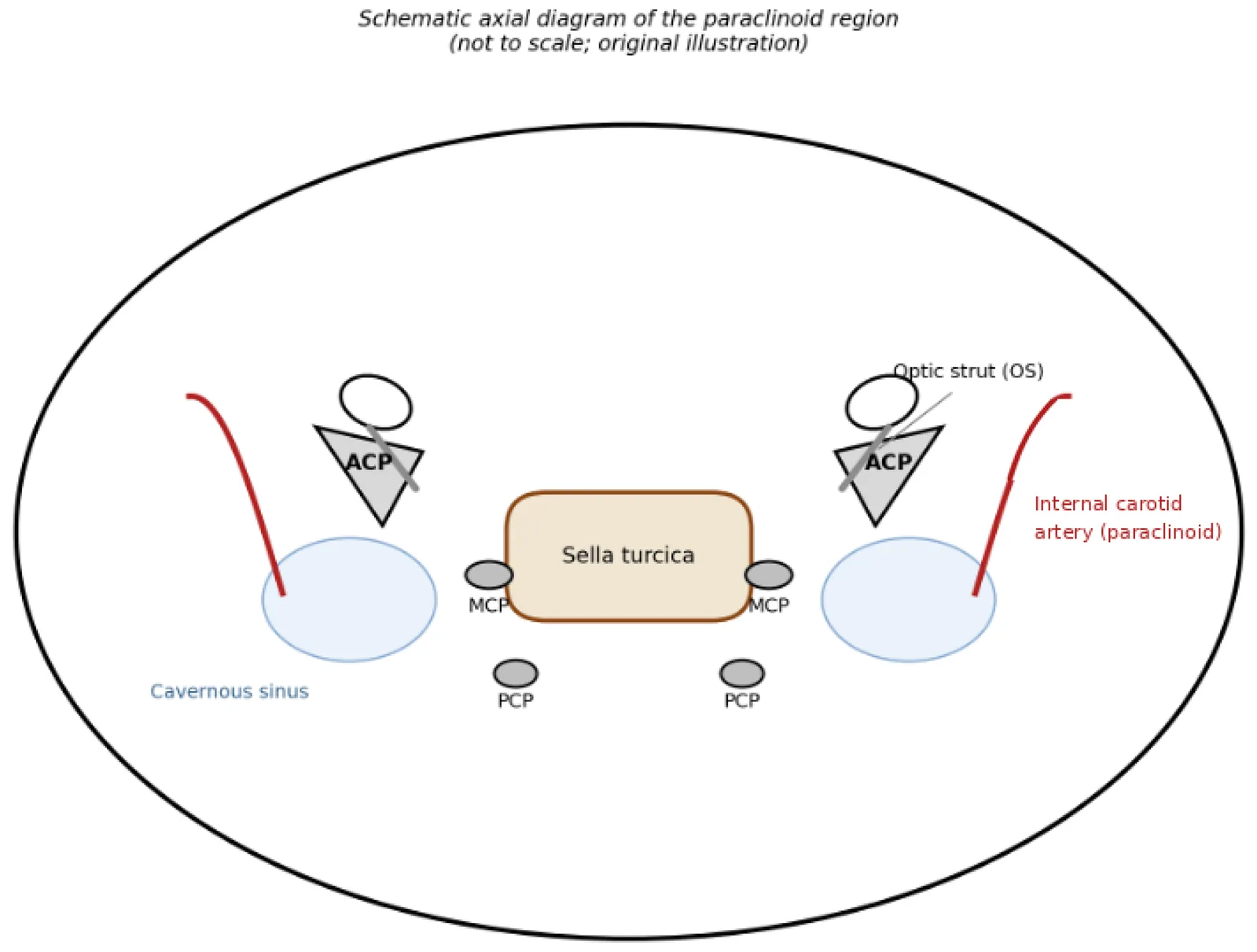
Schematic axial diagram of the paraclinoid region, illustrating the anterior clinoid process (ACP), middle clinoid process (MCP), posterior clinoid process (PCP), optic strut (OS), sella turcica, cavernous sinus, and the paraclinoid segment of the internal carotid artery. Original illustration prepared for this manuscript; not to scale and not derived from any copyrighted source. Gray lines indicate the optic struts (OS).

**Figure 2:**
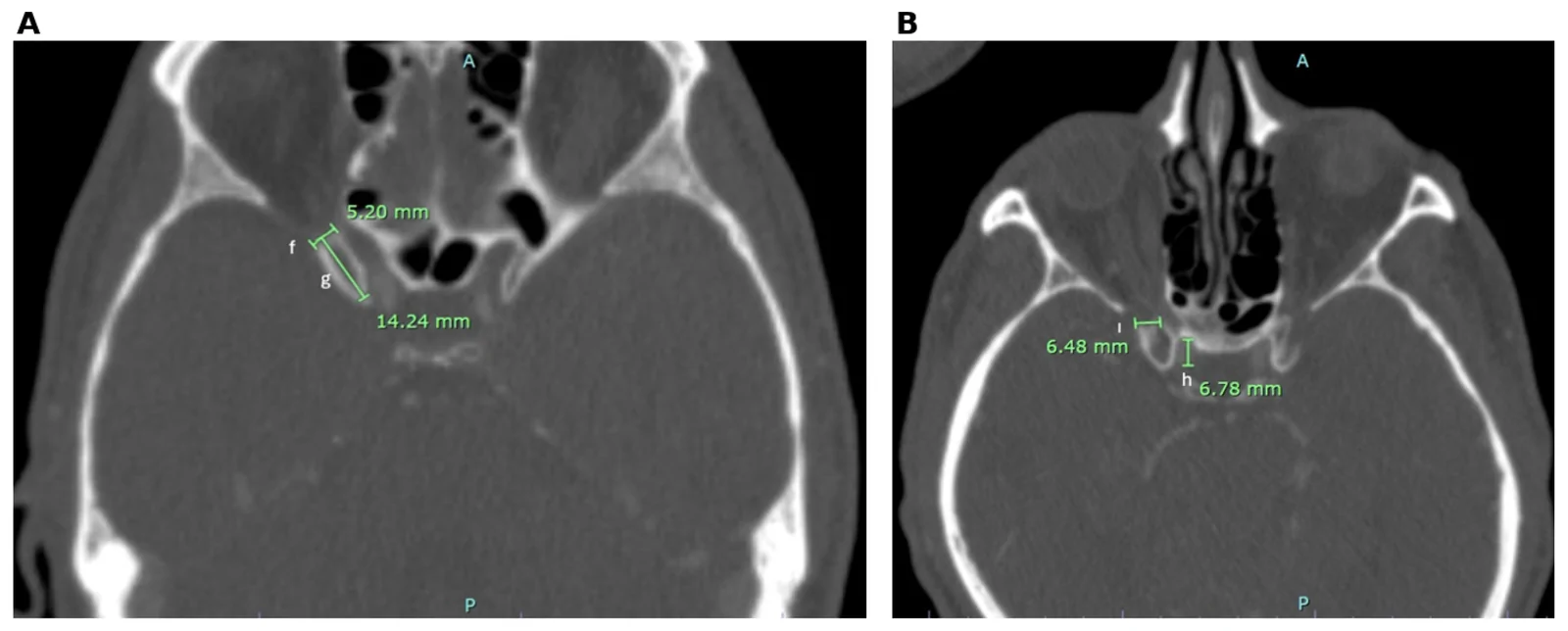
Morphometric measurements of the anterior clinoid process (ACP) and its relationship to the optic canal (CO) and optic strut (OS), reorganized as a labeled panel. (**A**) ACP basal width (f) and length (g). (**B**) Distance between the ACP apex and the posterior margin of the OS (h), and ACP basal width at the medial border of the CO (I). A and P on the images indicate the anterior and posterior orientation of the axial CT sections, respectively.

**Figure 3:**
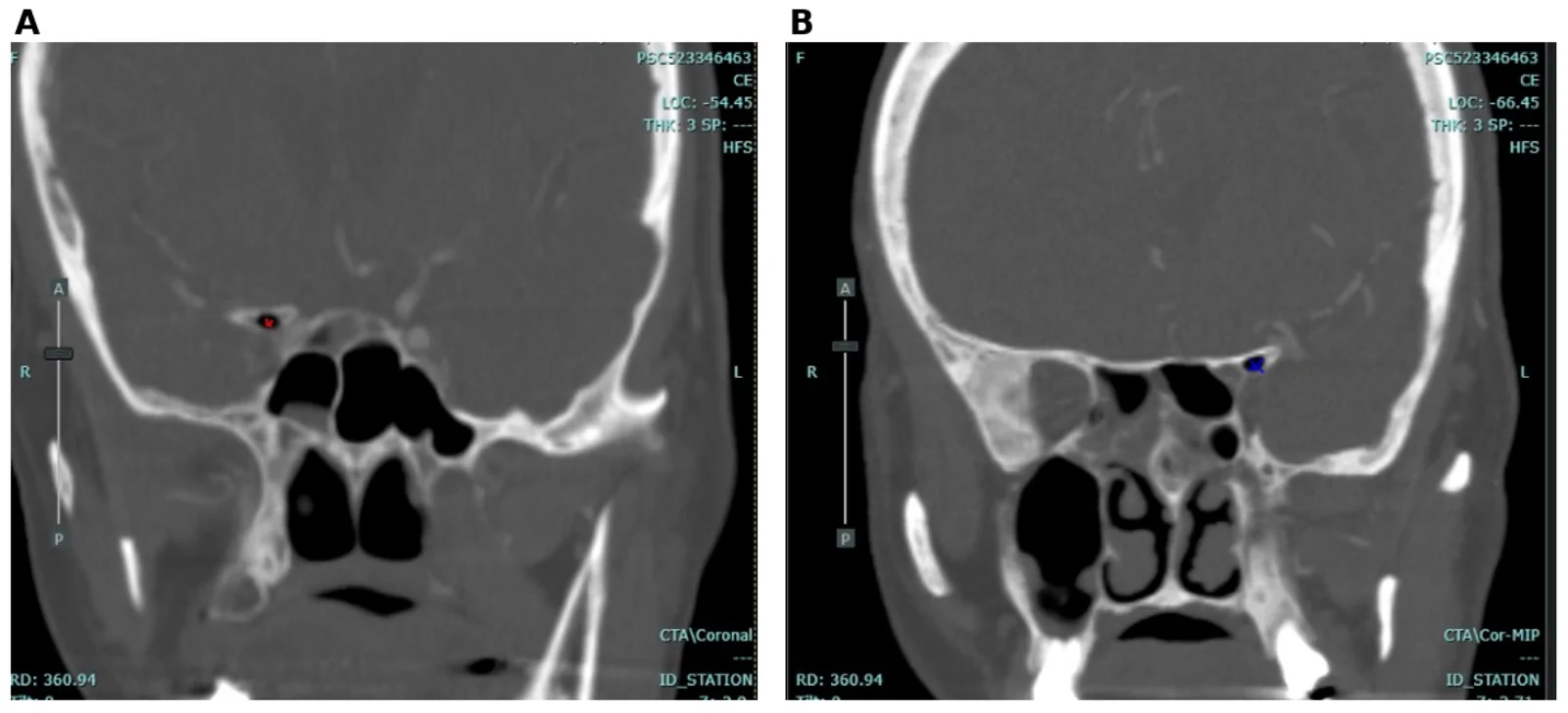
ACP pneumatization patterns. (**A**) Posterior pneumatization (Type 1) on the right side (red star indicates the pneumatized region). (**B**) Anterior pneumatization (Type 2) on the left side (blue star indicates the pneumatized region). “A”, “R”, “L”, and “P” indicate anatomical orientation markers (anterior, right, left, and posterior, respectively), while “F” denotes the feet (inferior) direction, as automatically generated by the CT scanner.

**Figure 4:**
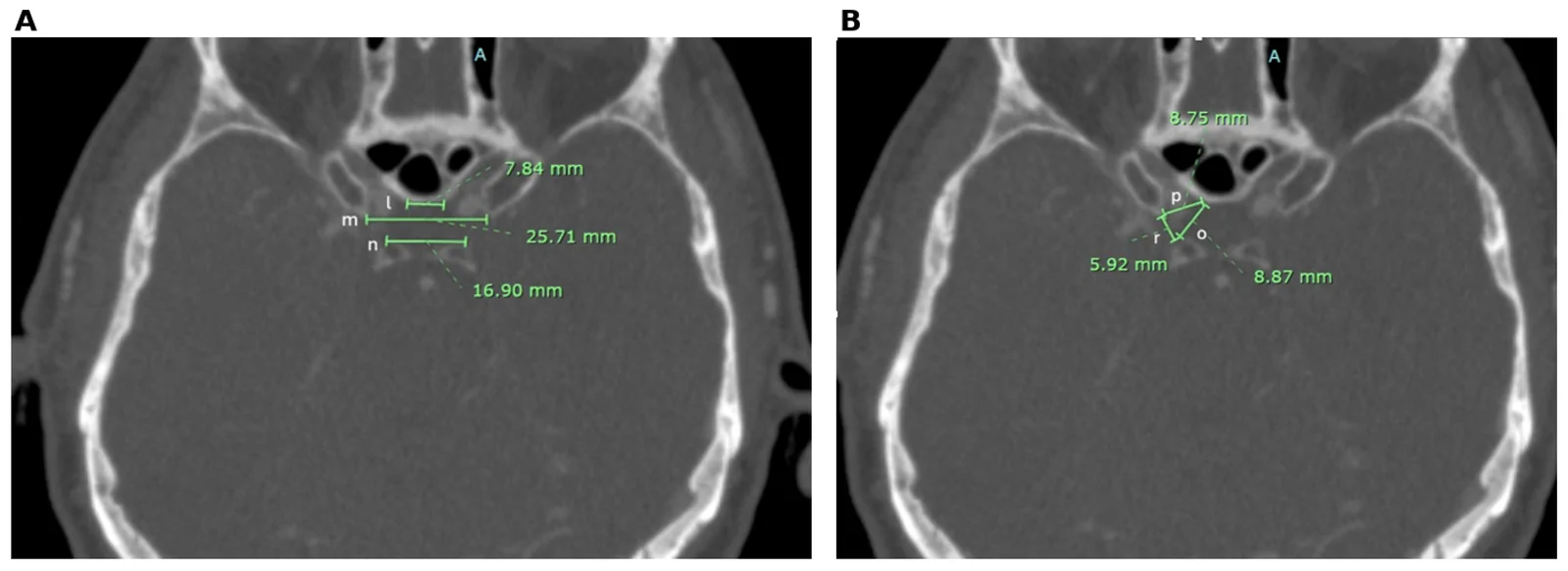
Interclinoid distances. (**A**) Bilateral distances between the ACPs (m), the MCPs (l), and the PCPs (n). (**B**) Ipsilateral (right-sided example) ACP–PCP (r), ACP–MCP (p), and MCP–PCP (o) distances. A on the image indicates the anterior orientation of the axial CT sections, respectively.

**Figure 5:**
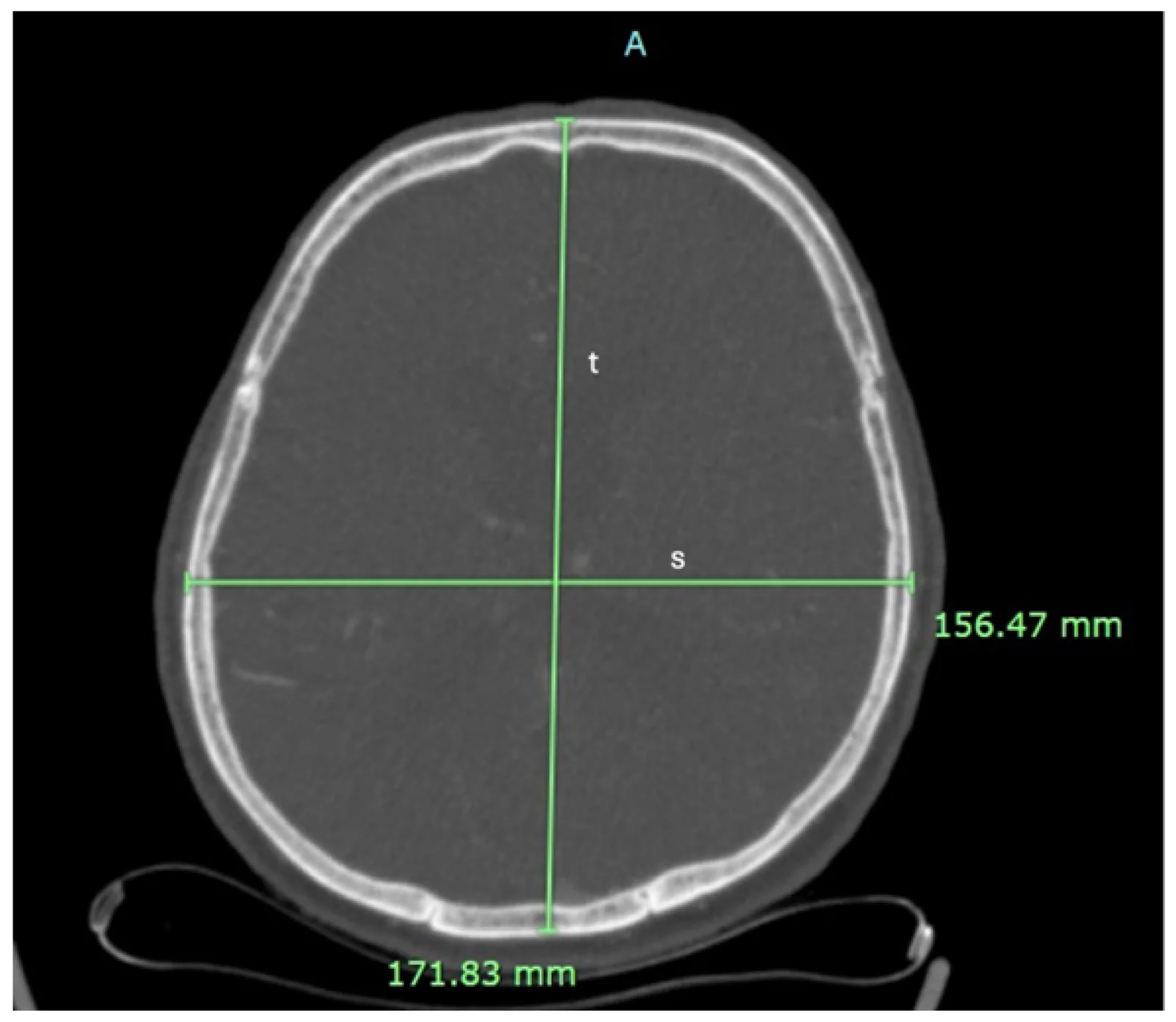
Biparietal distance (s) and fronto-occipital distance (t). A on the image indicates the anterior orientation of the axial CT section.

**Table 1:** Distribution of sex and ACP pneumatization types.

Category	Value	n	%
Sex	Male	59	57.8
	Female	43	42.2
ACP right	Type 0	81	79.4
	Type 1	7	6.9
	Type 2	9	8.8
	Type 3	5	4.9
ACP left	Type 0	84	82.4
	Type 1	7	6.9
	Type 2	8	7.8
	Type 3	3	2.9

**Table 2:** Descriptive statistics of ACP measurements (right vs. left).

Parameter	Right, Mean ± SD	Right Min	Right Max	Left, Mean ± SD	Left Min	Left Max
ACP basal width	4.51 ± 0.92	2.46	7.98	4.59 ± 0.80	3.00	7.00
ACP length	14.69 ± 2.56	8.55	22.86	14.90 ± 2.21	9.00	21.00
ACP–OS distance	5.31 ± 0.92	3.33	8.26	5.42 ± 0.80	3.00	8.00
ACP basal width at CO level	5.60 ± 0.99	2.58	7.94	5.48 ± 0.79	2.78	7.25
ACP–PCP distance	7.62 ± 1.80	2.00	14.00	7.97 ± 2.02	3.00	16.00
ACP–MCP distance	8.75 ± 1.85	5.00	18.00	9.47 ± 1.90	5.00	15.00
MCP–PCP distance	9.40 ± 2.09	4.00	18.00	9.40 ± 2.02	6.00	18.00

SD, standard deviation.

**Table 3:** Descriptive statistics of bilateral distances and cranial diameters.

Parameter	Mean ± SD	Min	Max
ACP–ACP bilateral distance	25.87 ± 2.23	20.00	31.00
MCP–MCP bilateral distance	8.73 ± 1.86	5.00	16.00
PCP–PCP bilateral distance	14.53 ± 2.57	10.00	21.00
Biparietal diameter	145.50 ± 6.89	129.00	160.00
Fronto-occipital diameter	171.64 ± 8.16	153.00	188.00

**Table 4:** Right-left comparison of ACP and interclinoid distances (overall and by sex).

Parameter	Overall, *p*	Male, *p*	Female, *p*
ACP basal width	0.399	0.436	0.728
ACP length	0.338	0.615	0.355
Basal width/length ratio	0.824	0.617	0.820
ACP–OS distance	0.197	0.735	0.071
ACP basal width at CO level	0.249	0.104	0.802
ACP–PCP distance	0.015 *	0.006 *	0.830
ACP–MCP distance	<0.001 **	0.004 *	0.015 *
MCP–PCP distance	0.984	0.761	0.624

* *p* < 0.05; ** *p* < 0.001.

**Table 5:** Right-left distribution of ACP pneumatization types (overall sample).

Right\Left	Type 0	Type 1	Type 2	Type 3	Total
Type 0	73 (71.6%)	3 (2.9%)	4 (3.9%)	1 (1.0%)	81 (79.4%)
Type 1	3 (2.9%)	3 (2.9%)	1 (1.0%)	0 (0.0%)	7 (6.9%)
Type 2	6 (5.9%)	0 (0.0%)	2 (2.0%)	1 (1.0%)	9 (8.8%)
Type 3	2 (2.0%)	1 (1.0%)	1 (1.0%)	1 (1.0%)	5 (4.9%)
Total	84 (82.4%)	7 (6.9%)	8 (7.8%)	3 (2.9%)	102 (100%)

McNemar–Bowker test: χ^2^ = 2.733, df = 6, *p* = 0.841 (male: χ^2^ = 4.200, df = 5, *p* = 0.521; female: χ^2^ = 2.400, df = 5, *p* = 0.791).

**Table 6:** Correlations between bilateral paraclinoid distances and cranial diameters.

Variable Pair	Overall (r)	Male (r)	Female (r)
ACP-ACP–MCP-MCP	0.352 **	0.514 **	0.193
ACP-ACP–PCP-PCP	0.269 **	0.316 *	0.215
ACP-ACP–Biparietal	0.409 **	0.354 **	0.383 *
ACP-ACP–Fronto-occipital	−0.062	−0.135	−0.116
MCP-MCP–PCP-PCP	0.274 **	0.340 **	0.193
MCP-MCP–Biparietal	0.214 *	0.346 **	0.136
MCP-MCP–Fronto-occipital	−0.058	−0.095	0.045
PCP-PCP–Biparietal	0.227 *	0.321 *	0.124
PCP-PCP–Fronto-occipital	0.052	0.003	0.104
Biparietal–Fronto-occipital	0.140	0.217	−0.151

* *p* < 0.05; ** *p* < 0.01 (Pearson correlation; n = 102).

**Table 7:** Distribution and right-left comparison of AMI, ASS, and PAI categories.

Index/Category	Right, n (%)	Left, n (%)	McNemar–Bowker χ^2^ Test (df = 6)
Anteroclinoid Morphometric Index (AMI)			*p* = 0.660
AMI-1 (linear type)	22 (21.6)	21 (20.6)	
AMI-2 (proportional type)	55 (53.9)	57 (55.9)	
AMI-3 (expansive type)	25 (24.5)	24 (23.5)	
ACP Size Score (ASS)			*p* = 0.280
ASS-1 (compact form)	25 (24.5)	18 (17.6)	
ASS-2 (intermediate form)	52 (51.0)	54 (52.9)	
ASS-3 (expansive form)	25 (24.5)	30 (29.4)	
Paraclinoid Access Index (PAI)			*p* = 0.226
PAI-1 (compressed corridor)	19 (18.6)	12 (11.8)	
PAI-2 (standard corridor)	73 (71.6)	81 (79.4)	
PAI-3 (expansive corridor)	10 (9.8)	9 (8.8)	

AMI, basal width/length ratio: AMI-1 ≤ 0.269; AMI-2, 0.27–0.35; AMI-3 ≥ 0.35. ASS, sum of ACP length, basal width, ACP–OS distance, and basal width at the CO level (right-side thresholds): ASS-1 ≤ 27.569; ASS-2, 27.57–32.65; ASS-3 ≥ 32.65. PAI, ACP–OS distance (midpoint thresholds): PAI-1 ≤ 4.49 mm; PAI-2, 4.5–6.5 mm; PAI-3 ≥ 6.5 mm.

**Table 8:** Post hoc correlations of ASS and PAI with the ipsilateral ACP–MCP distance and CO transverse diameter.

Correlation	Right (r, *p*)	Left (r, *p*)
ASS–ACP-MCP distance	r = 0.382, *p* < 0.001	r = 0.312, *p* = 0.001
PAI (ACP–OS distance)–ACP–MCP distance	r = 0.228, *p* = 0.021	r = 0.103, *p* = 0.304
ASS–CO transverse diameter	r = 0.180, *p* = 0.070	r = 0.232, *p* = 0.019
PAI (ACP–OS distance)–CO transverse diameter	r = 0.179, *p* = 0.072	r = 0.352, *p* < 0.001

Pearson correlation; n = 102. These analyses were performed post hoc and were not part of the original, prespecified analysis plan (see Section 2.5); they should be interpreted as hypothesis-generating.

## Data Availability

The data presented in this study are not publicly available due to ethical and privacy restrictions but are available from the corresponding author upon reasonable request.

## Supplementary Materials

The following supporting information can be downloaded at: https://www.mdpi.com/article/10.3390/tomography12100140/s1, Table S1: Exploratory correlation screen between the Anteroclinoid Morphometric Index (AMI), ACP Size Score (ASS), Paraclinoid Access Index (PAI), and all other measured interclinoid distances and cranial/optic canal diameters (Pearson correlation, n = 102).
