# Supplementary material for: Evaluation of Parameters Relevant to Paraclinoid Microsurgery: Morphometric Analysis of the Anterior, Middle, and Posterior Clinoid Processes
Source: Tomography. 2026 Sep 24;12(10):140. doi: 10.3390/tomography12100140 (PMC13709547; doi:10.3390/tomography12100140)
Supplement: Supplementary file 1 [file tomography-12-00140-s001.zip › Supplementary_Table_S1.pdf]

Supplementary Table S1. Exploratory correlation screen between the Anteroclinoid Morphometric Index (AMI), ACP Size Score (ASS), Paraclinoid Access Index (PAI), and all other measured interclinoid distances and cranial/optic canal diameters (Pearson correlation, n=102).

This supplementary table accompanies the manuscript "Evaluation of Parameters Relevant to Paraclinoid Microsurgery: Morphometric Analysis of the Anterior, Middle, and Posterior Clinoid Processes." As noted in the Methods (Section 2.5) and Discussion (Limitations), AMI, ASS, and PAI were screened against all measured distances and diameters in addition to the two relationships (ASS/PAI vs. ipsilateral ACP-MCP distance and CO transverse diameter) reported in the main text (Table 8). Correlations involving global cranial dimensions (biparietal and fronto-occipital diameter) are interpreted as reflecting generalized craniometric scaling — consistent with the correlations already reported between bilateral interclinoid distances and cranial diameters in Table 6 of the main text — rather than local paraclinoid topography, and are provided here for full transparency. * denotes $p<0.05$, uncorrected for multiple comparisons; given the number of comparisons performed (n=126), these should be interpreted as hypothesis-generating only.

Index	Target parameter	r	p	Sig.
AMI (right)	ACP-PCP distance (right)	-0.000	1.000	
AMI (right)	ACP-PCP distance (left)	-0.069	0.492	
AMI (right)	ACP-MCP distance (right)	-0.036	0.718	
AMI (right)	ACP-MCP distance (left)	-0.037	0.714	
AMI (right)	MCP-PCP distance (right)	-0.191	0.055	
AMI (right)	MCP-PCP distance (left)	-0.198	0.046	*
AMI (right)	ACP-ACP bilateral distance	+0.170	0.087	
AMI (right)	MCP-MCP bilateral distance	+0.018	0.858	
AMI (right)	PCP-PCP bilateral distance	+0.041	0.680	
AMI (right)	Biparietal diameter	+0.037	0.714	
AMI (right)	Fronto-occipital diameter	-0.285	0.004	*
AMI (right)	CO transverse diameter (right)	+0.105	0.294	
AMI (right)	CO transverse diameter (left)	+0.070	0.486	
AMI (right)	CO vertical diameter (right)	-0.023	0.821	
AMI (right)	CO vertical diameter (left)	+0.020	0.840	
AMI (right)	CO anterior diameter (right)	+0.182	0.067	
AMI (right)	CO anterior diameter (left)	+0.182	0.067	
AMI (right)	CO posterior diameter (right)	+0.168	0.092	
AMI (right)	CO posterior diameter (left)	+0.177	0.075	
AMI (right)	CO length (right)	-0.248	0.012	*
AMI (right)	CO length (left)	-0.241	0.015	*

Index	Target parameter	r	p	Sig.
AMI (left)	ACP-PCP distance (right)	-0.075	0.451	
AMI (left)	ACP-PCP distance (left)	+0.026	0.795	
AMI (left)	ACP-MCP distance (right)	-0.132	0.186	
AMI (left)	ACP-MCP distance (left)	-0.122	0.220	
AMI (left)	MCP-PCP distance (right)	-0.141	0.158	
AMI (left)	MCP-PCP distance (left)	-0.227	0.022	*
AMI (left)	ACP-ACP bilateral distance	+0.259	0.008	*
AMI (left)	MCP-MCP bilateral distance	+0.315	0.001	*
AMI (left)	PCP-PCP bilateral distance	+0.052	0.607	
AMI (left)	Biparietal diameter	+0.018	0.860	
AMI (left)	Fronto-occipital diameter	-0.301	0.002	*
AMI (left)	CO transverse diameter (right)	+0.094	0.345	
AMI (left)	CO transverse diameter (left)	+0.184	0.065	
AMI (left)	CO vertical diameter (right)	-0.071	0.476	
AMI (left)	CO vertical diameter (left)	+0.112	0.264	
AMI (left)	CO anterior diameter (right)	+0.004	0.965	
AMI (left)	CO anterior diameter (left)	+0.018	0.860	
AMI (left)	CO posterior diameter (right)	+0.077	0.443	
AMI (left)	CO posterior diameter (left)	+0.056	0.573	
AMI (left)	CO length (right)	-0.293	0.003	*
AMI (left)	CO length (left)	-0.357	<0.001	*
ASS (right)	ACP-PCP distance (right)	-0.156	0.118	
ASS (right)	ACP-PCP distance (left)	-0.164	0.099	
ASS (right)	ACP-MCP distance (right)	+0.382	<0.001	*
ASS (right)	ACP-MCP distance (left)	+0.202	0.042	*
ASS (right)	MCP-PCP distance (right)	-0.039	0.698	
ASS (right)	MCP-PCP distance (left)	-0.006	0.951	
ASS (right)	ACP-ACP bilateral distance	+0.234	0.018	*
ASS (right)	MCP-MCP bilateral distance	+0.002	0.988	
ASS (right)	PCP-PCP bilateral distance	+0.268	0.007	*
ASS (right)	Biparietal diameter	+0.261	0.008	*
ASS (right)	Fronto-occipital diameter	+0.064	0.521	
ASS (right)	CO transverse diameter (right)	+0.180	0.070	
ASS (right)	CO transverse diameter (left)	+0.158	0.114	
ASS (right)	CO vertical diameter (right)	+0.042	0.675	

Index	Target parameter	r	p	Sig.
ASS (right)	CO vertical diameter (left)	-0.055	0.582	
ASS (right)	CO anterior diameter (right)	+0.133	0.183	
ASS (right)	CO anterior diameter (left)	+0.193	0.052	
ASS (right)	CO posterior diameter (right)	+0.240	0.015	*
ASS (right)	CO posterior diameter (left)	+0.146	0.143	
ASS (right)	CO length (right)	+0.450	<0.001	*
ASS (right)	CO length (left)	+0.409	<0.001	*
ASS (left)	ACP-PCP distance (right)	-0.098	0.326	
ASS (left)	ACP-PCP distance (left)	-0.034	0.734	
ASS (left)	ACP-MCP distance (right)	+0.305	0.002	*
ASS (left)	ACP-MCP distance (left)	+0.312	0.001	*
ASS (left)	MCP-PCP distance (right)	+0.129	0.195	
ASS (left)	MCP-PCP distance (left)	+0.181	0.069	
ASS (left)	ACP-ACP bilateral distance	+0.248	0.012	*
ASS (left)	MCP-MCP bilateral distance	+0.017	0.867	
ASS (left)	PCP-PCP bilateral distance	+0.190	0.056	
ASS (left)	Biparietal diameter	+0.300	0.002	*
ASS (left)	Fronto-occipital diameter	+0.114	0.253	
ASS (left)	CO transverse diameter (right)	+0.243	0.014	*
ASS (left)	CO transverse diameter (left)	+0.232	0.019	*
ASS (left)	CO vertical diameter (right)	+0.147	0.141	
ASS (left)	CO vertical diameter (left)	+0.086	0.391	
ASS (left)	CO anterior diameter (right)	+0.273	0.006	*
ASS (left)	CO anterior diameter (left)	+0.222	0.025	*
ASS (left)	CO posterior diameter (right)	+0.358	<0.001	*
ASS (left)	CO posterior diameter (left)	+0.285	0.004	*
ASS (left)	CO length (right)	+0.345	<0.001	*
ASS (left)	CO length (left)	+0.523	<0.001	*
PAI (right)	ACP-PCP distance (right)	-0.012	0.902	
PAI (right)	ACP-PCP distance (left)	+0.048	0.630	
PAI (right)	ACP-MCP distance (right)	+0.228	0.021	*
PAI (right)	ACP-MCP distance (left)	+0.132	0.185	
PAI (right)	MCP-PCP distance (right)	-0.043	0.669	
PAI (right)	MCP-PCP distance (left)	-0.084	0.399	
PAI (right)	ACP-ACP bilateral distance	+0.353	<0.001	*

Index	Target parameter	r	p	Sig.
PAI (right)	MCP-MCP bilateral distance	+0.072	0.469	
PAI (right)	PCP-PCP bilateral distance	+0.191	0.055	
PAI (right)	Biparietal diameter	+0.295	0.003	*
PAI (right)	Fronto-occipital diameter	+0.108	0.279	
PAI (right)	CO transverse diameter (right)	+0.179	0.072	
PAI (right)	CO transverse diameter (left)	+0.267	0.007	*
PAI (right)	CO vertical diameter (right)	-0.032	0.753	
PAI (right)	CO vertical diameter (left)	-0.067	0.500	
PAI (right)	CO anterior diameter (right)	+0.295	0.003	*
PAI (right)	CO anterior diameter (left)	+0.063	0.532	
PAI (right)	CO posterior diameter (right)	+0.327	<0.001	*
PAI (right)	CO posterior diameter (left)	+0.044	0.658	
PAI (right)	CO length (right)	+0.219	0.027	*
PAI (right)	CO length (left)	+0.097	0.331	
PAI (left)	ACP-PCP distance (right)	-0.016	0.874	
PAI (left)	ACP-PCP distance (left)	+0.101	0.311	
PAI (left)	ACP-MCP distance (right)	-0.023	0.816	
PAI (left)	ACP-MCP distance (left)	+0.103	0.304	
PAI (left)	MCP-PCP distance (right)	+0.087	0.386	
PAI (left)	MCP-PCP distance (left)	+0.059	0.553	
PAI (left)	ACP-ACP bilateral distance	+0.263	0.008	*
PAI (left)	MCP-MCP bilateral distance	+0.179	0.072	
PAI (left)	PCP-PCP bilateral distance	+0.130	0.194	
PAI (left)	Biparietal diameter	+0.282	0.004	*
PAI (left)	Fronto-occipital diameter	+0.088	0.378	
PAI (left)	CO transverse diameter (right)	+0.291	0.003	*
PAI (left)	CO transverse diameter (left)	+0.352	<0.001	*
PAI (left)	CO vertical diameter (right)	+0.120	0.228	
PAI (left)	CO vertical diameter (left)	+0.047	0.641	
PAI (left)	CO anterior diameter (right)	+0.278	0.005	*
PAI (left)	CO anterior diameter (left)	+0.205	0.038	*
PAI (left)	CO posterior diameter (right)	+0.328	<0.001	*
PAI (left)	CO posterior diameter (left)	+0.182	0.067	
PAI (left)	CO length (right)	+0.187	0.060	
PAI (left)	CO length (left)	+0.197	0.047	*
